# Differentiation and Immunological Function of MDSC-Derived Dendritic Cells

**DOI:** 10.1055/s-0042-1756659

**Published:** 2022-12-21

**Authors:** Zequn Ding, Yan Zhang

**Affiliations:** 1 State Key Laboratory of Oncogenes and Related Genes, Renji-Med-X Stem Cell Research Center, Ren Ji Hospital, School of Biomedical Engineering, Shanghai Jiao Tong University, Shanghai, PR China; 2 Med-X Research Institute, School of Biomedical Engineering, Shanghai Jiao Tong University, Shanghai, China

**Keywords:** MDSC-DCs, BMDCs, antitumor activity

## Abstract

Dendritic cells (DCs) play a key role in initiating and regulating immune responses, and in addition to their roles in vivo, DCs are used as natural adjuvants for various tumor vaccines. In vitro, monocytes can be used to induce DCs, but in tumor patients, due to insufficient bone marrow hematopoiesis, extramedullary hematopoiesis and tumor-associated myeloid cells expand, and monocytes mainly exist in the form of myeloid-derived suppressor cells (MDSCs). The purpose of this experiment was to explore the differences in the differentiation and immune function of DCs induced by MDSCs in tumor patients. In a mouse model, we used normal mouse bone marrow cell-derived DCs as control cells, and in a tumor-bearing model, we induced MDSCs in the spleen to generate DCs (MDSC-DCs). Through flow cytometry, we found that the production of MDSC-DCs was significantly higher than that of control mice, and the secretion of interferon-γ of MDSC-DCs was significantly reduced. Through OVA antigen presentation experiments, we found that the antigen presentation ability of MDSC-DCs was significantly decreased. Through adoptive treatment of tumor-bearing mice cells, we found that the antitumor immune function of MDSC-DCs was significantly reduced. After that, we explored the mechanism of the decrease of immune function activity of MDSC-DCs. We determined that the surface markers of MDSC-DCs were changed by flow cytometry. Through flow sorting and RNA sequencing, we found that some pathways and key gene expression in MDSC-DCs were changed. In conclusion, this study found that the immune function of MDSC-DCs decreased and explored the mechanism of the decreased immune function activity.

## Introduction

Dendritic cells (DCs) are unique and powerful professional antigen-presenting cells that are important for the initiation and regulation of immune responses. 1 DCs are widely distributed in peripheral tissues, capture foreign or self-antigens, and rapidly process these antigens for delivery to secondary lymphoid organs. 2 DCs play a key role in initiating and regulating immune responses, and in addition to their role in vivo, DCs are used as natural adjuvants in various tumor vaccines. 3 At present, the related research studies on DCs and their clinical application are gradually deepened. The inoculation method of DC vaccine is mainly to take the peripheral blood of tumor patients, induce them to generate DC in vitro, load the antigen and activate it with a combination of toll-like receptors ligands and cytokines, and finally return it to the patient. 4

In recent years, immunotherapy has been widely used in tumor treatment. However, the accumulation of tumor cell mutations can cause tumor cells to secrete a variety of immunosuppressive factors, resulting in changes in the tumor microenvironment (TME) and tumor immunosuppression. 5 Myeloid-derived suppressor cells (MDSCs) represent a heterogeneous population of immunosuppressive myeloid cells, and MDSCs are abnormal in a variety of disease settings, such as autoimmune disease and cancer. 6 MDSCs are arguably the most important protective cells of the TME, the cornerstone of the immunosuppressive barrier that protects tumors from the patient's immune system and immunotherapy. 5

Under normal circumstances, DCs can be induced by monocytes. At present, there are many reports in the literature that granulocyte/macrophage colony-stimulating factor (GM-CSF) is used to induce myeloid cells in vitro to obtain DC cells. The most commonly used method is the use of GM-CSF in combination with interleukin-4 (IL-4). 7 8 In tumor patients, due to insufficient bone marrow hematopoiesis, extramedullary hematopoiesis, tumor-associated myeloid cell expansion, and accumulation of MDSCs in the spleen are found. 9 In the peripheral blood of tumor patients, most of them are precursor cells prepared from MDSCs as DC tumor vaccines. 10 It has long been demonstrated in animal models that the induction of tumor-secreted cytokines leads to extramedullary hematopoiesis in the spleen and accumulation of MDSCs in the spleen. 11 12 In this study, we constructed a 4T1-BALB/c tumor-bearing mouse model and used MDSCs from the spleen of tumor-bearing mice to induce DCs in vitro, and used this instead of PBMCs from tumor-bearing patients to induce DCs in vitro to investigate whether MDSCs from tumor-bearing mice could induce sufficient DCs. We named the MDSC-induced DCs as MDSC-DCs and explored whether their immune function was reduced compared with normal human–induced DCs, which may provide new data support and theoretical support for immunotherapeutic options such as DC vaccines.

## Results

### MDSCs Accumulate in the Spleen of Tumor-Bearing Mice

**Fig. 1:**
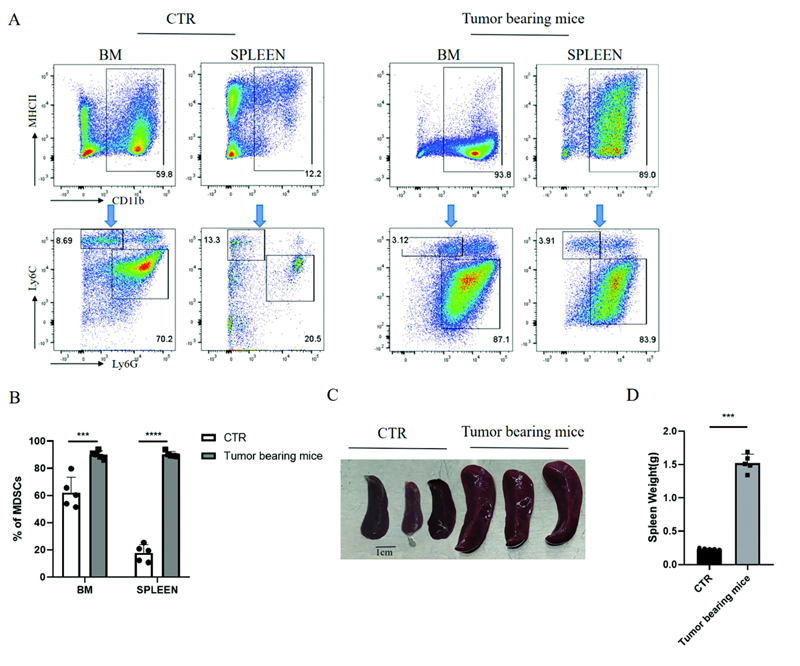
MDSCs accumulate in the spleen of tumor-bearing mice. ( **A** ) Representative flow cytometry plots of MDSCs in bone marrow cells and spleen cells of tumor-bearing mice and controls. ( **B** ) Statistical chart of the proportion of MDSCs in bone marrow cells and spleen cells of tumor-bearing mice and control group, *n* = 5 mice per group. ( **C** ) Representative pictures of the spleen of control and tumor-bearing mice. ( **D** ) Statistics of spleen weight of 30-day tumor-bearing mice and control mice of the same batch, *n* = 5 mice per group. * *p* < 0.05, ** *p* < 0.01, *** *p* < 0.001. Data were represented as mean ± SD. MDSC, myeloid-derived suppressor cell; SD, standard deviation.

Our study used the 4T1-BALB/c mouse tumor model to first identify the exact organ location of the accumulation of MDSCs, thereby utilizing this cell population for more in-depth studies. In mice, MDSCs are defined by the expression of CD11b+ Gr1+ and can be further divided into Ly6C ^−^ Ly6G ^+^ G-MDSCs and Ly6C ^+^ Ly6G ^−^ M-MDSCs (mononuclear-MDSCs). 13 We took the bone marrow cells and spleen cells of the tumor-bearing mice and the control group for flow analysis and found that MDSCs from the bone marrow and spleen of the tumor-bearing mice were expanded ( Fig. 1A ). After statistical analysis of the proportion of MDSCs, it was found that the content of MDSCs in the spleen was significantly higher than that in the control group ( Fig. 1B ). This suggests that MDSCs accumulate in the spleen of the tumor-bearing mice. Compared with the control group, the spleen of the tumor-bearing group was obtained through splenectomy and found that the spleen of the tumor-bearing group increased significantly in volume and weight ( Fig. 1C, D ). Based on the above results, we believe that MDSCs accumulate in the spleen of tumor-bearing mice.

### MDSCs Can Induce the Generation of DCs

**Fig. 2:**
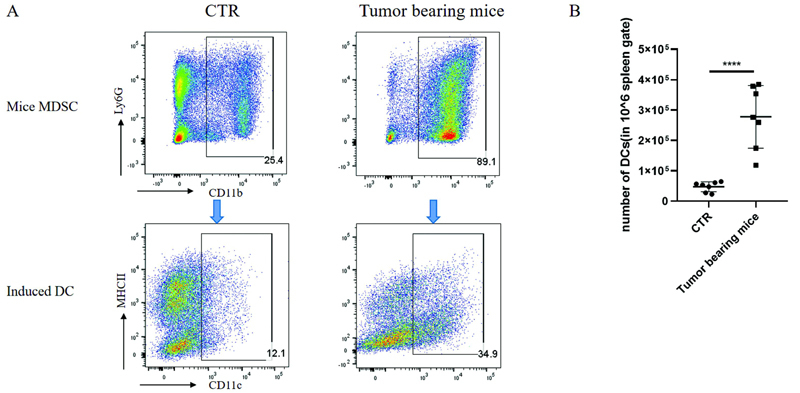
MDSCs can be induced to generate MDSC-DCs. ( **A** ) Representative flow cytometry plots of MDSCs in spleen cells of control mice and tumor-bearing mice and MDSC-DCs after induction of MDSCs for 6 days. ( **B** ) Quantitative analysis of the number of DCs induced by spleen cells in control mice and tumor-bearing mice per 106 cells, *n* = 7 mice per group. * *p* < 0.05, ** *p* < 0.01, *** *p* < 0.001. Data were represented as mean ± SD. DC, dendritic cell; MDSC, myeloid-derived suppressor cell; SD, standard deviation.

In the mouse model, we used spleen cells of 4T1-BALB/c mice bearing tumors for 30 days to replace the peripheral blood mononuclear cells of tumor patients, and bone marrow cells of normal mice to replace the peripheral blood of normal people, because the overall induction process is similar. The proportion of MDSCs (∼90%) in the spleen cells of tumor-bearing mice was significantly higher than that of normal control mice (∼25%). GM-CSF and IL-4 were added for in vitro induction, and tumor-bearing mice were induced to obtain MDSC-DCs. The differentiation efficiency was approximately 35%, while the normal control mice could hardly induce DCs ( Fig. 2A ). This result also confirms that under normal physiological conditions, MDSCs mainly exist in the bone marrow of mice instead of the spleen, and quickly differentiate into bone marrow cell-derived DCs (BMDCs). Flow quantitative analysis of the number of induced DCs per 10 ^6^ spleen cells showed that the number of MDSC-DCs induced by spleen cells of tumor-bearing mice was significantly higher than that of normal control mice ( Fig. 2B ). The above results confirmed that spleen cells of tumor-bearing mice could induce more DCs than normal mice.

### Decreased Antitumor Activity of MDSC-DCs

**Fig. 3:**
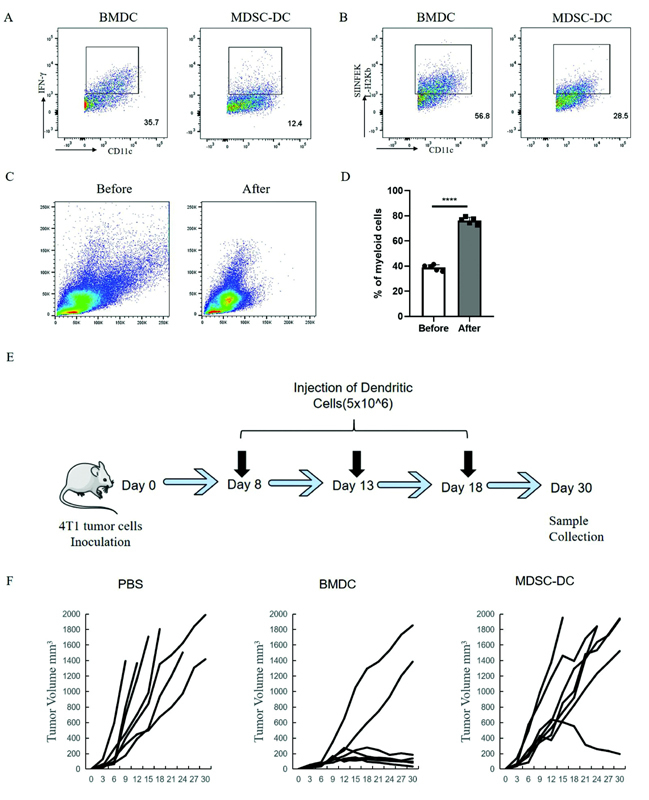
Decreased antitumor activity of MDSC-DCs. ( **A** ) Representative flow cytometry plots of IFN-γ secretion after 24-hour stimulation with LPS for BMDCs induced from normal mouse bone marrow and MDSC-DCs induced from tumor-bearing mouse spleen. ( **B** ) Representative flow cytometry plots of the proportion of SIINFEKL-H2Kb cells after BMDCs and MDSC-DCs were incubated with OVA for 48 hours. ( **C** ) Representative flow cytometry plots of MDSC-DCs before and after treatment with cell debris removal solution. ( **D** ) Percentage of viable myeloid cells before and after treatment with cell debris removal solution, *n* = 5 mice per group. ( **E** ) Flowchart of MDSC-DC vaccine preparation and treatment plan. ( **F** ) Tumor growth curves of 4T1 tumor-bearing mice treated with different groups of vaccines. Tumor size was measured every 3 days after vaccination. *n* = 7 mice per group. * *p* < 0.05, ** *p* < 0.01, *** *p* < 0.001. Data were represented as mean ± SD. BMDC, bone marrow cell-derived dendritic cell; DC, dendritic cell; IFN, interferon; LPS, lipopolysaccharide; MDSC, myeloid-derived suppressor cell; SD, standard deviation.

First, we measured the antitumor activity of MDSC-DCs in vitro. In the mouse model, we used the BMDCs induced by the control mice and the MDSC-DCs induced by the spleen of the tumor-bearing mice. To measure the nonspecific immune activity of MDSC-DCs, we induced and cultured BMDCs and MDSC-DCs in vitro, stimulated with lipopolysaccharide (LPS) on the 6th day to make the final concentration reach 100 ng/mL, stimulated for 24 hours, and detected interferon-γ (IFN-γ) secretion amount by flow cytometry; it was found that the IFN-γ secretion amount of MDSC-DCs was significantly reduced ( Fig. 3A ), which proved that the nonspecific immune function activity of MDSC-DCs was reduced. To test the antigen-presenting ability of MDSC-DCs, OVA antigen was added on the 5th day of in vitro induction and culture of normal mice and tumor-bearing mouse DCs. PE-CY7-conjugated OVA257–264 peptide SIINFEKL antibody was labeled for 15 minutes, and the antigen-presenting ability of MDSC-DCs was significantly lower than that of BMDCs, observed using flow cytometry ( Fig. 3B ). Therefore, the in vitro antitumor activity of MDSC-DCs is reduced.

To detect the antitumor activity of MDSC-DCs in vivo, we hope to prepare BMDC and MDSC-DC vaccines to treat tumor-bearing mice. For mature DC vaccines, antigens need to be prepared first to stimulate the maturation of DCs, and BMDCs are added to each 1 mL of MDSC-DC cell suspension. A 200 μL of tumor cell lysate to make the ratio of tumor cell lysate to MDSC-DCs 2:1, the mixed cell suspension was put into a constant-temperature cell incubator for 4 hours, then GM-CSF and LPS were added, and MDSC-DCs matured after 12 hours. Afterwards, the Debris Removal Solution was used to remove dead cells or cell debris generated during the culture of mature MDSC-DCs, and the cell debris after treatment was significantly reduced compared with that before treatment ( Fig. 3C ). The proportion of viable myeloid cells was also significantly increased after treatment with removal solution compared with before treatment ( Fig. 3D ).

After treating antigen-loaded mature MDSC-DCs and BMDCs with cell debris treatment solution, we performed adoptive cell therapy on 4T1-BALB/c tumor-bearing mice via tail vein injection of MDSC-DC and BMDC vaccine. The cell injection volume of MDSC-DCs and BMDCs was 5 × 10 ^6^ each time; phosphate-buffered saline (PBS) was used as the control group; and there were seven mice in each group. Vaccine treatments were administered via tail vein injection on days 8, 13, and 18, tumor size was measured every 3 days, tumor volumes were recorded, and tumor samples were collected on day 30 ( Fig. 3E ). The control group injected with PBS started to have dead individuals on the 12th day, the mice injected with the MDSC-DC vaccine began to have dead individuals from the 18th day, and the mice injected with the BMDC vaccine did not have any dead individuals until the 30th day, compared with BMDCs. Compared with the line trend graph of tumor volume in the MDSC-DC groups, the tumor volume of the mice injected with the BMDC vaccine was significantly down-regulated compared with the control group, while the MDSC-DC vaccine group showed a similar trend to the control group. It can be concluded that BMDCs can play a normal antigen-presenting function, and their vaccines can significantly inhibit tumors, while the antitumor immune activity of MDSC-DCs is low ( Fig. 3F ). Therefore, the in vivo antitumor activity of MDSC-DCs is reduced.

### Changes of Markers on the Surface of MDSC-DCs

**Fig. 4:**
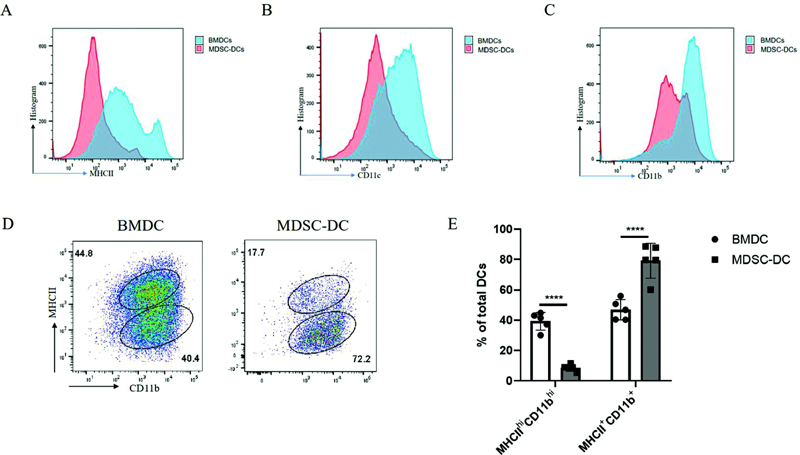
Changes of markers on the surface of MDSC-DCs. ( **A** ) Histogram comparison of MHCII expressions on the surface of MDSC-DCs induced by the spleen of tumor-bearing mice and bone marrow–induced BMDCs of normal mice. ( **B** ) Histogram comparison of CD11c expression on the surface of MDSC-DCs and BMDCs. ( **C** ) Histogram comparison of CD11b expression on the surface of MDSC-DCs and BMDCs. ( **D** ) Representative flow cytometry plots of the clustering of MDSC-DCs induced from the spleen of tumor-bearing mice and BMDCs induced from the bone marrow of normal mice. ( **E** ) The proportion of MHCII ^hi^ CD11b ^+^ and MHCII ^+^ CD11b ^+^ subpopulations of MDSC-DCs and BMDCs in total DCs, *n* = 5 mice per group. * *p* < 0.05, ** *p* < 0.01, *** *p* < 0.001. Data were represented as mean ± SD. BMDC, bone marrow cell-derived dendritic cell; DC, dendritic cell; MDSC, myeloid-derived suppressor cell; SD, standard deviation.

To further understand the difference between MDSC-DCs induced by spleen of tumor-bearing mice and BMDCs induced by bone marrow of normal mice, we detected some DC-related surface markers, such as MHCII, CD11c, and CD11b, by flow cytometry. Analysis was performed with FlowJo_V10 flow analysis software, and the histogram results showed that the expressions of MHCII, CD11c, and CD11b were significantly reduced on MDSC-DCs compared with BMDCs ( Fig. 4A–C ).

Culturing mouse bone marrow cells with GM-CSF has been widely used to generate CD11c ^+^ MHCII ^+^ cells resembling tissue DCs, commonly referred to as BMDCs. 14 Previously, by detecting DCs and macrophage-related markers and genes on the surface of BMDCs, a group of cells more similar to mature DCs, that is, a group of cells with higher MHCII expression, was named GM-DCs, while those more similar to macrophages were named GM-DCs. 15 We identified BMDC-related markers by flow analysis, and the results also divided into two subtypes, but they were not exactly the same as the two subtypes in the literature. We defined them as MHCII ^hi^ CD11b ^+^ and MHCII ^+^ CD11b ^+^ groups. The DCs induced by the spleen of tumor-bearing mice were typed, and it was found that the MHCII ^+^ CD11b ^+^ subgroup dominated, while the mature MHCII ^hi^ CD11b ^+^ subgroup with better antigen presentation function disappeared ( Fig. 4D ). We performed statistical analysis on the proportion of the two subgroups of BMDCs and MDSC-DCs in the total DCs, and found that the proportion of MHCII ^hi^ CD11b ^+^ subgroup of MDSC-DCs was significantly lower than that of BMDCs, while the proportion of MHCII ^+^ CD11b ^+^ group was significantly increased ( Fig. 4E ).

### The mTOR and PPAR Signaling Pathways of MDSC-DCs Are Inhibited

**Fig. 5:**
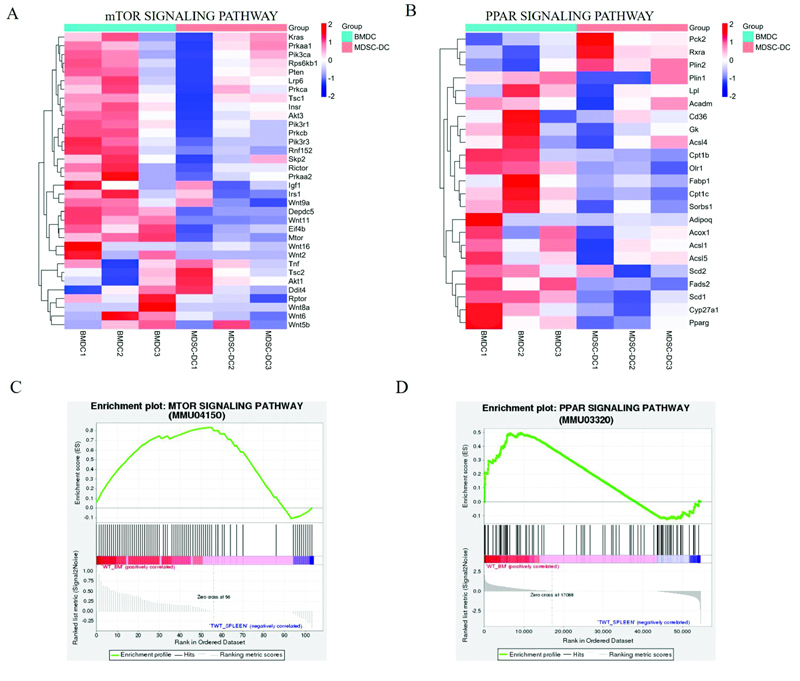
The mTOR and PPAR signaling pathways of MDSC-DCs are inhibited. ( **A** ) Heatmap of cluster analysis of BMDCs versus MDSC-DCs in the mTOR signaling pathway gene. *n* = 3 mice per group. ( **B** ) Heatmap of cluster analysis of BMDCs and MDSC-DCs in PPAR signaling pathway gene, *n* = 3 mice per group. ( **C** ) GSEA plot of MDSC-DCs compared with BMDCs in mTOR signaling pathway. ( **D** ) GSEA plot of MDSC-DCs compared with BMDCs in PPAR signaling pathway. BMDC, bone marrow cell-derived dendritic cell; DC, dendritic cell; GSEA, Gene Set Enrichment Analysis; MDSC, myeloid-derived suppressor cell; mTOR, mammalian target of rapamycin.

mTOR is a particularly critical regulator of DC differentiation, maturation, and function. 16 17 We sorted the BMDCs derived from the bone marrow of control mice and the MDSC-DCs derived from the spleen of tumor-bearing mice in vitro by flow sorting, and named them as BMDC and MDSC-DC, respectively, during RNA sequencing. Clustering analysis of BMDCs and MDSC-DCs in the genes of mTOR signaling pathway found that tumor suppressor factors such as Pten, Tsc1, Ddit4, mtor, Rptor, and other genes involved in controlling the activity of rapamycin complex 1 (mTORC1) were found in MDSCs-DCs and are all down-regulated. The known mTORC1-regulated transcription factors include PPAR-γ, STAT3, and HIF. We performed cluster analysis on the two types of DCs in PPAR signaling pathway. Through gene clustering heat map analysis, it was found that the PPAR signaling pathway genes of MDSC-DC were down-regulated as a whole compared with BMDC, including the adipogenic genes Scd1, Scd2, Plin1, Acsl1, Acsl5, lipid metabolism-related genes Fads2, Adipoq, Lpl, Gk, immune-related genes Cpt1b, Cd36, Rxra, proliferation-related gene Plin2, and encoding PPARγ protein gene Pparg ( Fig. 5A, B ). GSEA (Gene Set Enrichment Analysis) analysis of MDSC-DCs and BMDCs in the mTOR signaling pathway and PPAR signaling pathway showed that the gene enrichment of BMDCs in the two pathways was up-regulated compared with MDSC-DCs as a whole ( Fig. 5C, D ). This indicated that the mTOR signaling pathway and PPAR signaling pathway were inhibited in MDSC-DCs.

### Transformation of MDSC-DCs toward Granulocytes

**Fig. 6:**
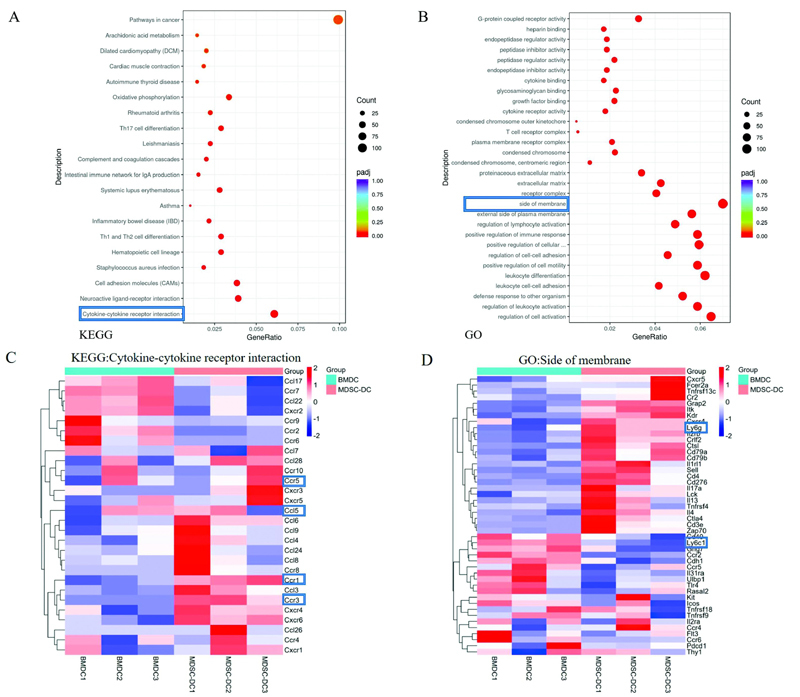
Transformation of MDSC-DCs toward granulocytes. ( **A** ) The bubble plot of the significant pathways of KEGG differences between BMDCs and MDSC-DCs. ( **B** ) The bubble plot of the significant pathways of GO differences between BMDCs and MDSC-DCs. ( **C** ) Heat map of common chemokines and their receptor genes screened in cytokine–cytokine receptor interaction in the KEGG pathway. ( **D** ) Heat map of selected genes related to chemokine and its receptor pathway in Side of membrane of GO pathway. BMDC, bone marrow cell-derived dendritic cell; DC, dendritic cell; MDSC, myeloid-derived suppressor cell.

The BMDCs induced from the bone marrow of control mice and the MDSC-DCs induced from the spleen of tumor-bearing mice in vitro were sorted by a flow sorter and were named BMDCs and MDSC-DCs, respectively, during RNA sequencing. Through bioinformatics analysis of transcriptome, we compared the KEGG pathway differences between BMDCs and MDSC-DCs. The bubble chart showed that the pathway difference of cytokine–cytokine receptor interaction was the most significant. Comparing BMDCs and MDSC-DCs, the GO pathway with the most significant difference was selected. Through the bubble chart analysis, it was found that the side of membrane pathway had the most significant difference ( Fig. 6A, B ).

Selecting the more common chemokines and their receptors in the cytokine–cytokine receptor interaction pathway, it can be found that the gene expressions of chemokines and their receptors in MDSC-DCs of tumor-bearing mice are generally up-regulated compared with BMDCs; especially, CCL5 and its receptors CCR1, CCR3, and CCR5 were all up-regulated. The genes related to chemokines and their receptor pathways were selected from the significantly different GO pathway side of membrane. Through gene clustering heat map analysis, it was found that Ly6G in MDSC-DC cells was significantly higher than that in BMDC cells, while Ly6C was significantly lower ( Fig. 6C, D ). CD11b ^+^ Ly6C ^hi^ Ly6G ^–^ marks immature monocytes morphologically and phenotypically resembling monocytes (M-MDSCs), and CD11b ^+^ Ly6C ^low^ Ly6G ^+^ marks immature polymorphonuclear cells morphologically and phenotypically resembling neutrophils (PMN- MDSC), so we believe that MDSC-DCs have a tendency to transform toward granulocytes.

## Discussion

In this study, we found that MDSCs accumulated in the spleen of tumor-bearing mice, and tumor-bearing mice were able to induce more DCs, which we named MDSC-DCs. MDSC expansion has been recognized as an important pathophysiological principle in most types of cancer and other diseases associated with chronic inflammation. In patients with advanced melanoma, the persistence of high levels of monocytic MDSCs has been reported to be associated with disease progression, poor survival, and poor response to checkpoint inhibitor therapy targeting PD-1 and CTLA-4. 18 19 In this experiment, the spleen of tumor-bearing mice and the control group were taken for flow cytometry, and the results showed that the proportion of MDSCs in tumor-bearing mice was significantly higher than that in normal mice, which was consistent with previous reports.

DC-based immunotherapy is safe and induces antitumor immunity, even in patients with advanced disease. However, clinical responses to DC vaccines have not been satisfactory. 20 In this study, the MDSC-DC vaccine prepared in our animal model test was infused back into mice and confirmed that the immunological activity of MDSC-DCs was reduced.

Afterwards, we explored the differences between the immunophenotype and genotype between MDSC-DCs induced by tumor-bearing mice and normal induced DCs, and explored the mechanism of the decreased immune function activity of MDSC-DCs. We found that compared with BMDCs induced from normal mice, the subtypes of MDSC-DCs with better antigen-presenting ability and closer to mature DCs were reduced in tumor-bearing mice-induced MDSC-DCs. In contrast, DC maturation is characterized by the upregulation of MHC class II co-stimulatory molecules on the cell surface and the production of various cytokines that can shape innate and adaptive immunity. 21 Therefore, we believe that the poor immune function of MDSC-DCs is related to the reduction of MHCII ^hi^ CD11b ^+^ subsets.

Many literatures have confirmed that the mTOR pathway plays an important role in the differentiation and function of DCs. Michael Haidinger and others cultured Mo-DC, GM-CSF, and IL-4 in peripheral blood mononuclear cells for 7 days and found that the level of mTOR pathway protein was specifically up-regulated, and inhibition of mTORC1 could reduce the survival rate of Mo-DC, and significantly hindered its immunostimulatory phenotype. 22 It has also been reported that mTORC1 is essential for MHC-II silencing during DC maturation. Tsc1 inhibits mTORC1 activation in DCs to ensure MHC-II expression on DCs, which is required for antigen presentation and CD4 T cell activation. 23 The RNA sequencing results of this experiment showed that the key genes of the mTOR signaling pathway were down-regulated in MDSC-DCs. GSEA analysis found that the mTOR signaling pathway was inhibited in MDSC-DCs. PPARγ is a key regulatory transcription factor of mTORC1, and PPARγ has a functional role in regulating immune responses through DCs. Many literatures have reported that PPARγ affects DC function by activating genes involved in lipid transport, metabolism, and presentation 24 25 ; our RNA-sequencing results showed that MDSC-DCs have overall down-regulated PPAR signaling pathway key genes compared with BMDCs, especially lipid metabolism and immune-related genes. Our study confirmed that both mTOR and PPARγ affect the function of MDSC-DCs, but whether the two pathways influence each other needs further exploration.

With bioinformatics transcriptome analysis of BMDC and MDSC-DC cells, we also selected significantly different KEGG pathway cytokine–cytokine receptor interaction and GO pathway side of membrane genes for comparative analysis, and found that MDSC. Compared with BMDCs, the gene expression of chemokines and their receptors in DCs was up-regulated as a whole, especially CCL5 and its receptors CCR1, CCR3, and CCR5 were up-regulated. The genes related to chemokine and its receptor pathway in the GO pathway side of membrane were analyzed by gene clustering heatmap analysis. Compared with BMDC cells, Ly6G was significantly increased in MDSC-DC, while Ly6C was significantly decreased. This suggests that the monocyte-like cells of MDSC-DCs have a tendency to transform into granulocytes. M-MDSCs can promote a pool of tumor-associated DCs by differentiating into inflammatory DCs (inf-DCs), which appear to have specific phenotypes and are key components of antitumor responses. 26 The chemokine CCL5 was shown to play an important role in granulocyte and monocyte differentiation, 27 and we hypothesized that inhibition of CCL5 in tumor-bearing mice might reverse the monocyte-to-granulocyte differentiation trend, thereby enhancing MDSC- The immune function of DCs, but the specific mechanism needs to be further explored.

In conclusion, this study found that the immune function of MDSC-DCs decreased, and explored the mechanism of the decreased immune function activity, which provided new data and theoretical support for clinical DC vaccine and other immunotherapy programs.

## Materials and Methods

### Cell Lines and Mice

The 4T1 mammary carcinoma cell line purchased from the American Type Culture Collection was used. All cells were cultured in RPMI 1640 (Gibco, United States) supplemented with 10% fetal bovine serum (Excel, China) at 37°C under 5% CO _2_ .

BALB/c female mice (6–8 weeks old) were obtained from Shanghai Jisijie Laboratory Animal Co., Ltd., China. A total of 10 ^6^ tumor cells were injected subcutaneously into the hind legs of mice to construct a 4T1-BALB/c mouse tumor model. All animal experiments were performed in accordance with the regulations of the Laboratory Animal Welfare and Ethics Committee of Shanghai Jiao Tong University.

### In Vitro Induction of BMDCs and MDSC-DCs

BMDCs were isolated from leg bones of BALB/c mice, and splenocytes were isolated from spleen of tumor-bearing mice to induce MDSC-DCs. The above cells were cultured in RPMI1640 medium containing 10% FBS, 10 ng/mL IL-4, and GM-CSF. Cultured in complete medium at 37°C with 5% CO _2_ for 7 days, supplemented with fresh RPMI, GMCSF, and IL-4 cultured cells on day 3, and cultured BMDCs and MDSC-DCs on day 7 using a cell scraper to scrape off the cells. as the starting material for subsequent experiments.

### DC Antigen Uptake Assay

On the 5th day of culturing DCs, 40 μg/mL OVA257–264 peptide (Thermo Fisher Scientific) was added, and after 48 hours of culture, they were labeled with PE-CY7-conjugated OVA257–264 peptide SIINFEKL antibody (Thermo Fisher Scientific) for 15 minutes, and the expression level of OVA was detected by flow cytometry.

### Flow Cytometry

The cells were resuspended in PBS to 10 ^7^ /mL, and 0.5 μL of fluorescent antibody was added to the cell suspension for flow antibody staining. The cell suspension was placed on ice and incubated in the dark for 30 minutes. Multiparametric analysis was performed on a Fortessa analyzer (BD Biosciences) and data were analyzed using FlowJo-V10 software (Tree Star, Ashland, Oregon, United States).

### Preparation of DC Vaccine

A 200 μL of tumor cell lysate was added to each 1 mL of MDSC-DC suspension, so that the ratio of tumor cell lysate to MDSC-DCs was 2:1. The cells were seeded into a six-well plate, GM-CSF and LPS were added, mixed well, and then incubated overnight in a 5% CO _2_ 37.5°C incubator. The cells were gently scraped and suspended with a cell scraper the next day. Cell debris was removed with a cell debris removal solution (Miltenyi Biotec), purified cells were extracted by Ficoll density gradient centrifugation, and the cells were diluted to 10 ^7^ /mL.

### Adoptive Therapy of Tumor-Bearing Mice

4T1 mice bearing tumors for 7 days were treated by tail vein injection of MDSC-DCs and BMDCs, and the injection volume of MDSC-DCs and BMDCs was 5 × 10 ^6^ cells each time, and PBS was used as a control group. Injections were given on days 8, 13, and 18, tumor size was measured every 3 days, tumor volumes were recorded, and tumor samples were collected on day 30.

### BMDCs and MDSC-DCs Sorting and RNA Sequencing

The BMDCs and MDSC-DCs induced in vitro were collected with a cell scraper into a 50 mL sterile centrifuge tube, and PBS was added to resuspend the cells to 10 ^7^ cells/mL. Then 2 μL of flow antibody was added to 1 mL of cell suspension and incubated in the dark for half an hour. Cell populations of interest were sorted by flow cytometry (BD Biosciences). TRIzol Reagent (MRC) was added and blew evenly until no obvious cell clumps are visible at the bottom of the centrifuge tube, and the NEB Next PolyA mRNA Magnetic Separation Module (New England Biolabs, Ipswich, Massachusetts, United States) was used to complete mRNA separation. RNA-Seq libraries were prepared using the Kappa Strand RNA-Seq Kit from Illumina (Kappa Biosystems, Wilmington, Massachusetts, United States). The RNA-seq library was sequenced on Illumina HiSeq platform with paired-end 2 × 150 as the sequencing mode.

### Statistical Analysis

All data were analyzed by GraphPad Prism 7, the paired *t* -test was used to analyze the significance between the two groups, and the one-way analysis of variance rate was used to compare the significance between the multiple groups. All data are presented as mean ± standard deviation. When *p* -value <0.05, the results were considered statistically significant, and *p* -value <0.001 was considered to be extremely significant.
